# Complete Genome Sequences of Endophytic Bacilli Isolated from Grapevine Plants

**DOI:** 10.1128/MRA.01265-19

**Published:** 2019-11-27

**Authors:** Andrey V. Mardanov, Elena P. Chizhevskaya, Alexander M. Lazarev, Andrey L. Rakitin, Alexey V. Beletsky, Vladimir K. Chebotar, Nikolai V. Ravin

**Affiliations:** a Institute of Bioengineering, Research Center of Biotechnology, Russian Academy of Sciences, Moscow, Russia; b All-Russia Research Institute of Agricultural Microbiology, St. Petersburg, Russia; c All-Russia Research Institute of Plant Protection, St. Petersburg, Russia; Queens College

## Abstract

The endophytic strains Bacillus amyloliquefaciens V417 and V167 were isolated from cultured grape plants. We sequenced the complete genomes of these strains to reveal their potential beneficial properties for plant growth promotion and control of fungal pathogens. Genes responsible for the synthesis of antimicrobial compounds and siderophores were identified.

## ANNOUNCEMENT

Endophytic bacterial strains were isolated from vegetative tissues of wine grape variety Fetiaska cultivated in the south of Russia. The shoots were washed with distilled water, rinsed in 70% ethanol, and placed for 10 min in 10% hydrogen peroxide. After sterilization, the material was washed three times with sterile water. Pieces of xylem and the core of the shoot were laid out on the surface of agarized R2A (1) medium and cultivated at 28°C for 5 days. Pure cultures were obtained from colonies and grown in LB medium at 30°C overnight. Two isolated strains, V417 and V167, were identified, using 16S rRNA gene analysis, as members of the genus *Bacillus*.

The total cellular DNA was isolated using the cetyltrimethylammonium bromide (CTAB)-NaCl method (2). The complete genomes were sequenced using the pyrosequencing and Oxford Nanopore platforms. Shotgun genome libraries for pyrosequencing were prepared using the GS FLX Titanium rapid library preparation kit (Roche, Switzerland) and amplified using the GS Titanium LV emulsion PCR (emPCR) kit (Lib-L) v.2. The libraries were sequenced on the Roche GS FLX instrument using the GS FLX Titanium sequencing kit XL+. For strain V417, pyrosequencing generated 349,127 single-strand reads with an average length of 286 bp; the reads were assembled into 149 contigs with an *N*_50_ value of 161,082 bp using the Newbler assembler v.3.0 (454 Life Sciences, Branford, CT). For strain V167, a total of 580,157 single-strand reads with an average length of 301 bp were obtained, and the reads were assembled into 153 contigs (*N*_50_, 339,802 bp). In addition, genomic DNAs were sequenced on a MinION platform (Oxford Nanopore) using ligation sequencing kit 1D and FLO-MIN106 cells. Nanopore sequencing generated 28,637 reads with an average length of 6,455 bp for strain V417 and 71,046 reads with an average length of 9,088 bp for strain V167. Nanopore reads were mapped on the contigs using Burrows Wheeler Aligner (BWA) v.0.7.15 (3), and the contigs were merged using npScarf (4). Finally, pyrosequencing reads were mapped on the npScarf contigs using Bowtie 2 v.2.3.4.1 (5), and the assembled sequence was polished using Pilon v.1.22 (6). Complete sequences of circular chromosomes were assembled; plasmids were not found. Gene search and annotation were performed using the Rapid Annotations using Subsystems Technology (RAST) server (7). Screening for secondary metabolite-related genes was performed using the Web server antiSMASH v.5.0.0 (8). BWA v.0.7.15 was used with parameter -x (read type) set to ont2d, and default settings were used for the rest of the software.

The main characteristics of the genomes of strains V417 and V167 are shown in Table 1. Both strains were assigned to Bacillus amyloliquefaciens. The average nucleotide identity (ANI), calculated using ANICalculator v.1.0 (9), between the genomes of these strains and *B. amyloliquefaciens* subsp. *plantarum* CC178 (10) was 99.97 to 99.98%, and the ANI between the V417 and V167 genomes was 99.96%. Genome analysis of strains V417 and V167 revealed the presence in each genome of gene clusters responsible for the synthesis of antimicrobial compounds and siderophores, including surfactin, plantazolicin, macrolactin, bacillaene, fengycin, difficidin, bacillibactin, and bacilysin.

**TABLE 1:** Main characteristics of the *B. amyloliquefaciens* V417 and V167 genomes

Parameter	Value for strain:	
	V417	V167
Genome size (bp)	3,907,893	3,904,474
GC content (%)	46.5	46.4
No. of protein-coding genes	4,060	3,980
No. of tRNA genes	82	74
No. of rRNA operons	9	7

### Data availability.

The complete genome sequences of *B. amyloliquefaciens* V417 and V167 have been deposited in GenBank under the accession numbers CP044359 and CP044360, respectively. The versions described in this paper are the first versions (CP044359.1 and CP044360.1). The raw sequences have been deposited in the Sequence Read Archive under the accession numbers PRJNA572567 and PRJNA572568.
